# A Case of Petechiœ Sine Febre

**Published:** 1791

**Authors:** Samuel Ferris

**Affiliations:** Physician in London.


### VII. *A Case of Petechiæ sine Febre. Communicated in a Letter to Dr.* Simmons *by* Samuel Ferris, *M. D. F. A. S. Physician in London.*

*To Dr.* Simmons.

Dear Sir,

AGREEABLY to my promise, I communicate to you the history of a case which fell under my observation in January last: a case of *Petechiæ sine Febre.* This is either a disease of so rare occurrence, that but few practitioners have had an opportunity of seeing it; or if many have seen it, but few of the many have given any description of it to the Public. Two or three only of my medical friends know any thing of the complaint from their own experience. From one of them, Mr. Rumsey, a very assiduous and observing practitioner at Amersham in Buckinghamshire, I have received notes of several cases, which occurred to him in practice, in his own neighbourhood. As for myself, I never saw but two instances of it before that, the history of which you now receive, and those I saw at Edinburgh about eight years ago.

Dr.

Dr. Graff publiſhed an Inaugural Diſſertation on this diſeaſe, at Gottingen, in 1775; and he is conſidered as the firſt who wrote expreſſly on the ſubject, although Lazarus Riverius is referred to as having alluded to this diſeaſe in his chapter *de febre peſtilenti*—(ſee "Lazari Ri-"verii praxeos medicæ Lugduni editæ, 1653," p. 348.)

Since the publication of Dr. Graff's Theſis, the hiſtory of a ſimilar caſe has been publiſhed in the Tranſactions of the Medical Society of Copenhagen, and Dr. Duncan, of Edinburgh, has publiſhed ſome account of this diſeaſe in a volume of medical caſes and obſervations; and two years ago Dr. Adair, jun. from having obſerved ſeveral caſes of it, likewiſe in Edinburgh, made it the ſubject of his very ingenious Inaugural Theſis.

Riverius affixed no particular appellation to this diſeaſe to diſtinguiſh it from thoſe petechial complaints which have fever as eſſential to their exiſtence. Dr. Graff diſtinguiſhed it by the name *petechiæ ſine febre*; this name conveys no idea of the effuſion of blood, which, from ſome ſource or other, generally accompanies an appearance of petechiæ without fever, and therefore Dr. Duncan propoſed to call this diſeaſe *petechianoſos* vel *aimorrhœa*; and in order to connect the idea of both occurrences, Dr. Adair calls it *hæmorrhœa petechialis*: But neither the names propoſed by Dr. Duncan, nor that adopted by Dr. Adair, are expreſſive of the abſence of fever; and I, on that account, prefer the name, under which, Dr. Graff firſt deſcribed the diſeaſe: for the abſence of fever is one of its moſt ſtriking peculiarities.

### C A S E.

M. C. daughter of J. C. a publican, in Hart-ſtreet, Bloomſbury, was brought to me, January 20th laſt; ſhe was ſeven years old in December, 1790; ſhe had always appeared to be a healthy, and ſhe was always an active child. She had never been ſubject to any particular complaints, nor had ſhe been in the leaſt indiſpoſed for three years prior to the appearance of the diſeaſe which was the occaſion of my ſeeing her: and three years before, ſhe had only a ſlight remittent fever, which the apothecary, who accompanied the child to my houſe, informed me, was not of very long continuance. Her food had ever been as good, and her clothing and habits ſuch as the children of people in the ſituation of her father and mother are uſually accuſtomed to, and they are reſpectable people, in their line of life.

During the night of the 17th of January laſt, ſome bright red ſpots appeared on her left foot, and on both of her legs: from that night to the 20th, when I firſt ſaw her, many more made their appearance on different parts of her body, gradually increaſing in number from the legs upwards, and on the 20th there were ſeveral on her arms, neck, and face; thoſe on the thighs were the largeſt, and many of them were livid, like bruiſes; ſome likewiſe on the neck and cheek were livid, and there was one larger than a ſhilling on the inſtep of the left leg, livid, hard, and ſomewhat prominent, as if blood had been effuſed into the cellular membrane, and had coagulated there.

On the morning of the 18th, her mother, when ſhe went to the child's bedſide, obſerved that her noſe had bled a little during the night, and that ſome dark-coloured clotted blood came from her mouth. Her mother then firſt noticed the ſpots on her legs. During the night of the 18th, when the child fell aſleep, her mother ſaw her noſe bleed, and, as it appeared to her, more than it had bled the night before, and ſome blood, partly clotted, and partly fluid, came likewiſe from her mouth, of a dark colour. The child herſelf did not ſeem reſtleſs; but her mother, from apprehenſion, leſt the blood ſhould ſuffocate her, awakened her repeatedly, and immediately as ſhe was awakened, the blood ceaſed to flow.

No hæmorrhage occurred through the day of the 18th nor of the 19th, nor in the night of the 19th did the noſe bleed, as it had done before; but ſo ſoon as ſhe was aſleep, blood began to flow, and ſome clots likewiſe came from the mouth, as in the preceding night, but in greater quantity, and always ſtopping immediately, whenever ſhe was awakened. She had a very trifling cough on the morning of the 20th, but had not any before, nor even the leaſt ſenſible indiſpoſition of any kind prior to the deſcribed affections obſerved on the morning of the 18th. Her appetite had been uniformly good, and her bowels regular, and were ſo on the 20th; nor had her water been obſerved to change in the leaſt from its uſual appearance. She had ſlept well, had been conſcious of no increaſe of heat, and her ſkin had been, and was, when I ſaw her, ſoft and natural to the touch.

On the 20th, when ſhe was brought to me for my advice, ſhe ſeemed perfectly alert, not in the leaſt debilitated; her pulſe was calm and regular, and by no means weak; but her tongue was a very little white, and, as her mother informed me, it had been more ſo during the night, and in the morning before I ſaw her. She was not in the leaſt unuſually thirſty; her fauces were tumefied, and the ſwoln amygdalæ were livid, and ſeemingly ruptured, for there was the appearance of coagulated blood, of a dark colour, hanging about the ſurface of them, as on the ſurface of an ill-conditioned ulcer; her breath was exceſſively offenſive, but was never noticed to have been ſo before the morning of the 18th. Her gums were clean and ſound. She felt no uneaſineſs from ſwallowing, nor did ſhe complain of the leaſt pain in any part of her body.

My preſcription on the 20th was the following:

℞ Decocti Corticis Peruviani ℥j

Pulveris ejuſdem, gran. x.

Syrupi Papaveris albi,

Tincturæ Radicis Serpentariæ Virginienſis, ana ʒj.

Miſce fiat hauſtus quater inter horas xxiv. ſumendus.——Let her eat oranges *ad libitum*.

The

The notes which I took of her ſituation, and the directions I gave afterwards, were as follow:

January 21ſt. The ſpots are nearly as yeſterday, but ſome are now obſervable on the edge of the tongue, on the gums, and one on the *tunica ſclerotica* of the right eye. She had no bleeding from the noſe, and leſs from the mouth laſt night; her appetite is not ſo good as yeſterday; ſhe complains of pain in the right hypogaſtric region. She has ſtill a little cough; her urine of laſt night was whitiſh, that of this morning is of a deep red colour, as if ſtrongly tinged with blood. Her pulſe is quicker and rather lower; her tongue ſomewhat white.

The draughts and oranges to be continued, and let her drink wine and water as her common beverage.

22. She ſlept well laſt night, and but little blood came from her mouth. Her noſe did not bleed; her appetite is not ſo good; the ſpots ſeem to diminiſh; the pain in the hypogaſtric region continues; her urine is ſtill red, but her mother ſays it is of a lighter tinge. Her pulſe is not very quick, but low; her tongue as before.

Let ten drops of the acid elixir of vitriol be added to each draught.

23d. The ſymptoms are nearly as yeſterday; more *vibices* appear on the thighs, legs, and other parts of the body; and very ſlight preſſure on the arms occaſions them. No blood flowed from the mouth laſt night; her urine in conſiderable quantity, and a little tinged with blood. Her tongue is whitiſh; ſhe had two ſtools yeſterday.—The ſame medicines to be continued.

24. The ſmall petechial ſpots are diminiſhed; and ſeveral are entirely gone; the *vibices* remain as yeſterday; the urine of laſt night is of a bright florid blood colour; that of this morning darker, with light red ſediment, but nothing like coagulum, nor has ſhe made ſo much water during the laſt twenty-four hours, as ſhe made in the twenty-four preceding. She ſlept well, and but very little blood came from the mouth; the fauces are ſtill tumefied, but the amygdalæ are leſs livid. The pain of the hypogaſtric region continues; ſhe ſometimes refers it to the ſeat of the right kidney. She had no motion yeſterday; her pulſe is quick and low; her tongue a little furred, but moiſt; ſhe was ſomewhat more than uſually thirſty yeſterday, but was not ſenſible of the leaſt extraordinary heat or dryneſs of the ſkin.

Five

Five grains of the Powder of Bark to be added to each draught.

25th. She ſlept well laſt night; no blood has flowed from the mouth; her breath is leſs offenſive; the petechial ſpots are on the decline, but the *vibices* remain on the legs and arms; her urine has been in leſs quantity than before, and leſs tinged with blood. She had one motion yeſterday; her pulſe is nearly as yeſterday; her tongue and mouth clean; her appetite mended, and ſhe is in good ſpirits.

27th. Every ſymptom is abating; her urine is returning to its natural appearance.

29th. She is ſtill better than on the 27th; the petechial ſpots are gone; her urine is nearly natural.

31ſt. She ſeems in every reſpect well. The urine of the 29th, has depoſited a little ſediment, with ſome few particles of the colour of blood. Yeſterday and to-day her urine has been natural; but there are ſtill very ſlight traces of *vibices* remaining; her appetite and ſpirits are exceedingly good; her throat is well; her breath not the leaſt offenſive; and her bowels are regular.

Her draughts to be continued morning and evening for a few days.

There is nothing more ufual than for phyficians to attempt to explain the fource and caufes of the different phenomena of difeafes. But the theoretical opinions of different phyficians, concerning thefe circumftances, are frequently fo various and contradictory, that we need require no clearer elucidation to prove the general inutility of fuch attempts.

Under fuch a conviction, it would be inconfiftent in me to enter into a long difcuffion of the *Ratio Symptomatum* of *Petechiæ fine Febre.*

It can fcarcely be denied that the blood circulating through the veffels of the living body, is thinner in its texture at one time than at another. Riverius, Dr. Duncan, and others, have confidered a preternatural thinnefs of the blood as the immediate fource of the *petechiæ* and *vibices* apparent in the difeafe above defcribed. It has been fuppofed alfo by fome phyfiologifts that it is poffible for fuch a partial laxity of the vafcular fyftem to exift, as to occafion an eafy rupture of the finer veffels, from any very flightly applied force, or to admit of the blood's paffage through parts not permeable by the blood when fuch parts are in their natural ftate, and that without any perceptible diminution of vital energy, or at leaft fufficient to interrupt the full exertion of mufcular aƈtion. Such a ſtate of laxity has been likewiſe conſidered as the ſource of the *petechiæ* and *vibices* in the diſeaſe juſt now deſcribed; and the remarkable circumſtance of the blood ceaſing to flow, as mentioned in the above caſe, when the child was awakened, is a ſtrong preſumptive proof of the juſtneſs of ſuch an inference. For as there is leſs occaſion for muſcular exertion, during ſleep, there is conſequently leſs energy excited, and all the aſſumed conſequences of ſuch a ſuppoſed laxity might be expeƈted to happen, whenever the vital energy ſhould diminiſh as it does in ſleep, and to be ſubverted whenever that energy ſhould be rouſed for the due performance of muſcular motion.

But perhaps the faƈt is, that both the aſcribed cauſes of thinneſs of blood, and partial laxity of veſſels, may aſſiſt in the produƈtion of *petechiæ ſine febre*; a diſeaſe ſeemingly very much allied in its nature to the true ſcurvy.

I have the honour to be, &c.

SAMUEL FERRIS.

*John Street, Bedford Row,*
April 6, 1791.

VIII.

